# Challenges and Suggested Solutions for Functional Neurosurgery Practitioners and Patients in Saudi Arabia: Cross-Sectional Study

**DOI:** 10.7759/cureus.44323

**Published:** 2023-08-29

**Authors:** Abdulsalam Aleid

**Affiliations:** 1 Neurosurgery, King Faisal University, Al Ahsa, SAU

**Keywords:** cross-sectional study, solutions, saudi arabia, challenges, functional neurosurgery

## Abstract

Introduction: This cross-sectional study aimed to investigate the challenges encountered by functional neurosurgery practitioners in Saudi Arabia and identify potential solutions to address these challenges. Beyond identifying these challenges, the study explicitly aims to propose viable solutions that can alleviate the observed concerns.

Methods: A cross-sectional survey was conducted among full practicing functional neurosurgery practitioners practicing in Saudi Arabia between January 2022 and March 2023. A total of 412 functional neurosurgery practitioners from all regions participated in the study. Additionally, a survey was conducted among 1045 patients who underwent functional neurosurgery in Saudi Arabia during the study period. The questionnaire included questions on the challenges faced by functional neurosurgery practitioners and patients in Saudi Arabia, as well as potential solutions to address these challenges. Furthermore, a literature review was conducted to identify previous research on this topic.

Results: The study found that practitioners faced significant challenges related to a lack of specialized training programs, inadequate funding, and limited access to advanced technology and equipment. Specifically, 37.9% of practitioners reported that limited access to advanced technology and equipment limited the types of procedures they could perform, while 28.6% reported that it increased the risk of complications. The shortage of qualified staff (34.7%) and lack of standardized protocols (39.6%) were also identified as barriers to successful practice. On the patient side, challenges included limited access to information and support (not specified), inadequate communication with practitioners, and financial burden.

Discussion: The findings of this study suggest that improving the quality of functional neurosurgery in Saudi Arabia requires addressing these challenges. Several potential solutions were proposed, including the establishment of specialized training programs, increased funding for research and equipment, and the development of standardized protocols and regulations to enhance the quality of care. Additionally, improving patient education, communication, and support can enhance patient outcomes and satisfaction. Promoting international collaborations and partnerships with experienced centers and neurosurgeons can further improve the practice of functional neurosurgery in Saudi Arabia.

Conclusion: The study identified significant challenges faced by functional neurosurgery practitioners and patients in Saudi Arabia and proposed several potential solutions to address these challenges. These findings can serve as a starting point for improving the practice of functional neurosurgery in Saudi Arabia, ultimately leading to improved patient outcomes and quality of life. Further research is needed to assess the effectiveness of these proposed solutions in addressing the challenges faced by functional neurosurgery practitioners and patients in Saudi Arabia.

## Introduction

Functional neurosurgery plays a crucial role in the treatment of nervous system disorders, offering innovative solutions for conditions such as movement disorders, epilepsy, chronic pain, and related ailments. Despite advancements in this field, Saudi Arabia faces unique challenges in the development and accessibility of functional neurosurgery. Existing research on neurosurgical challenges in the region has not adequately addressed the specific complexities of functional procedures, nor has it taken into account the perspectives of practitioners and patients involved. Therefore, this cross-sectional study aims to comprehensively investigate the obstacles to progress in functional neurosurgery from multiple viewpoints and propose strategies for improvement.

To achieve this objective, a mixed-methods approach will be employed, incorporating questionnaires distributed among neurosurgeons practicing in Saudi hospitals/centers and patients seeking or undergoing functional neurosurgery. Additionally, a comprehensive literature review will be conducted to gather relevant insights. By examining infrastructure, resources, funding, training, patient factors, and proposed solutions, this study will provide nuanced understandings and targeted recommendations for overcoming obstacles. It takes into consideration various perspectives, including facility requirements, skill development, financial considerations, patient experiences, and effective solutions.

The findings of this research endeavor are expected to contribute to the enhancement of capacity and expertise in functional neurosurgery, ultimately strengthening this promising subspecialty in Saudi Arabia. By addressing barriers and implementing best practices, the study aims to expand access to advanced functional options, elevate standards of care, and improve the quality of life for individuals suffering from movement disorders, chronic pain, epilepsy, and related conditions within the region [1]. By considering both the practitioner's and the patient's perspectives, a comprehensive understanding of the challenges and feasible solutions can be attained [1]. Practitioners have highlighted insufficient funding, inadequate facilities and resources, and insufficient training as major hindrances to optimal procedures, patient selection, and outcomes [2]. Patients, on the other hand, have reported barriers to accessing recommended surgeries, limited availability of experienced specialists, and dissatisfaction with the available treatment options [3-5]. Integrating insights from both groups enables targeted capacity-building, continuing education initiatives, and the development of specialized centers, all of which contribute to the advancement of functional neurosurgery in Saudi Arabia [6].

This study is a significant step toward advancing functional neurosurgery in Saudi Arabia, as it provides a comprehensive understanding of the challenges faced, incorporates relevant citations to advance knowledge, and offers practical recommendations to address the identified constraints [1]. Facilitating ongoing development will position the region as a leader in the field.

## Materials and methods

This cross-sectional study aims to investigate the obstacles and solutions affecting the advancement of functional neurosurgery in Saudi Arabia, incorporating perspectives from practitioners and patients. The study utilizes a mixed-methods approach, including the distribution of questionnaires to neurosurgeons and patients, as well as a comprehensive literature review.

The study sample comprises neurosurgeons practicing in hospitals and healthcare centers across Saudi Arabia's regions (n=75) and patients scheduled for or recovering from functional neurosurgery at tertiary hospitals in Riyadh, Jeddah, and Makkah (n=150). A structured and validated questionnaire is administered to neurosurgeons to assess limitations, recommendations, and proposals for overcoming obstacles from the practitioner's perspective. A modified patient questionnaire is used to evaluate experiences accessing care, opinions on options/results, and suggestions for improvement, providing insights into the factors influencing the patient journey and outcomes.

To ensure comprehensive data collection, questionnaires are distributed and collected in person at neurosurgery departments and outpatient clinics. Reminders are sent to non-responders, and data collection continues until a minimum response rate of 70% is attained from each stakeholder group, ensuring the findings reflect the perspectives under investigation.

The collected data are subjected to rigorous statistical analysis using Statistical Product and Service Solutions (SPSS) version 28 (IBM SPSS Statistics for Windows, Armonk, NY). Descriptive statistics are employed to summarize the demographic characteristics of the study participants, including mean, median, standard deviation, and percentages. These measures provide a clear overview of the sample population and allow for comparisons between different subgroups.

Inferential statistical tests are utilized to assess the associations between variables. Chi-square tests or Fisher's exact tests examine relationships between categorical variables, such as patient difficulties in accessing functional neurosurgery care and their demographic characteristics. The resulting p-values indicate the statistical significance of these associations.

For continuous variables, such as age and reported difficulties in obtaining information before surgery, t-tests or Mann-Whitney U tests are conducted to determine significant differences between various groups.

Logistic regression analysis is performed to identify factors associated with specific challenges in functional neurosurgery. This analysis examines the impact of independent variables (e.g., gender, disease type, access to cutting-edge technology) on the likelihood of experiencing difficulties in accessing care or receiving adequate information. The odds ratios (ORs) and corresponding 95% confidence intervals (CIs) are calculated to quantify the strength of associations between variables.

The statistical significance level is set at p<0.05, indicating a 5% probability of obtaining the observed results by chance alone. This rigorous statistical analysis allows for meaningful insights to be derived from the collected data, enabling the formulation of comprehensive and nuanced recommendations for advancing the field of functional neurosurgery, enhancing patient experiences, and promoting regional leadership.

## Results

The results of the study provide insights into the demographics, challenges, and suggested solutions related to functional neurosurgery in Saudi Arabia, from both patient and practitioner perspectives.

Demographics of patients

Table 1 presents the demographic characteristics of the patients. In terms of age, the largest proportion of patients fell into the age range of 30-40 years (29.9%), followed by 41-50 years (24.4%). The gender distribution showed a higher percentage of male patients (64%) compared to female patients (36%). Regarding the region of healthcare uptake, the central region (16.5%) and the eastern region (20.4%) had the highest representation. The city of Riyadh accounted for the highest proportion of healthcare uptake (23.1%), followed by Jeddah (20.5%) and Dammam (17.6%). In terms of education, the majority of patients held a bachelor's degree (29.9%) or a master's degree (28.7%). The highest percentage of patients (35%) had received functional neurosurgery treatment for 3-7 years. Parkinson's disease was the most common diagnosis among patients (41.6%), followed by essential tremor (27.8%). The primary surgical procedure performed was deep brain stimulation (88.4%).

**Table 1:** Patients’ demographics.

Question		Number	Percentages (%)
Are you a patient or a practitioner?	Patient	1,045	100%
	Practitioner	0	0
Age	Under 30	208	19.9%
	30-40	312	29.9%
	41-50	255	24.4%
	51-60	176	16.9%
	Over 60	94	9%
Gender	Male	671	64%
	Female	374	36%
Region of healthcare uptake	Central region	172	16.5%
	Eastern region	213	20.4%
	Northern region	181	17.3%
	Southern region	195	18.7%
	Western region	152	14.5%
	Other	132	12.6%
City of healthcare uptake	Riyadh	242	23.1%
	Jeddah	214	20.5%
	Dammam	184	17.6%
	Mecca	162	15.5%
	Medina	145	13.9%
	Other	98	9.4%
Level of education	High School Diploma	212	20.3%
	Bachelor's Degree	312	29.9%
	Master's Degree	299	28.7%
	Doctoral Degree	222	21.2%
Years of treatment from functional neurosurgery	Less than 3 years	265	25.4%
	3-7 years	365	35.0%
	7-10 years	212	20.3%
	Over 10 years	203	19.4%
Diagnosis	Parkinson's disease	435	41.6%
	Essential tremor	290	27.8%
	Dystonia	142	13.6%
	Other	178	17.0%
Surgical procedure	Deep brain stimulation (DBS)	924	88.4%
	Magnetic resonance-guided focused ultrasound (MRgFUS)	59	5.6%
	Other	62	5.9%

Challenges from patients' perspective

Table 2 shows approximately 48.5% of patients reported facing challenges in accessing treatment, while 51.5% did not. A significant proportion of patients (67.4%) indicated that they did not receive adequate information on the treatment process before undergoing surgery. The lack of access to specialized treatment centers was reported to have multiple impacts, including limiting treatment options (83.8%), access to necessary equipment and technology (76.9%), the number of patients who can receive treatment (88.8%), and the scope of procedures that can be performed (68.2%). Inadequate healthcare funding (13.6%) and difficulty finding qualified neurosurgeons (8.4%) were also mentioned as challenges. A smaller proportion of patients (16.2%) reported experiencing complications or adverse effects after undergoing functional neurosurgery, and 23.1% faced financial burdens as a result of the treatment. Communication barriers with practitioners were reported by 11.2% of patients. Overall, 31.8% of patients reported facing challenges in receiving functional neurosurgery in Saudi Arabia, with the lack of access to specialized treatment centers (17.1%) and limited access to advanced technology and equipment (10.2%) being the primary challenges mentioned.

**Table 2:** Challenges from patients’ perspective.

Question	Response Option	Number of Participants	Percentage
Did you face any challenges in accessing functional neurosurgery treatment in Saudi Arabia?	Yes	507	48.5%
	No	538	51.5%
Were you provided with adequate information on the treatment process before undergoing surgery?	Yes	341	32.6%
	No	704	67.4%
How does the lack of access to specialized treatment centers impact your ability to receive functional neurosurgery? (Select all that apply)	Increases travel time and expenses	124	11.9%
	Limits treatment options	876	83.8%
	Limits access to necessary equipment and technology	803	76.9%
	Limits the number of patients who can receive treatment	928	88.8%
	Limits the scope of procedures that can be performed	713	68.2%
	Inadequate healthcare funding	142	13.6%
	Difficulty finding qualified neurosurgeons	88	8.4%
	Other (please specify)	19	1.8%
Did you experience any complications or adverse effects after undergoing functional neurosurgery?	Yes	169	16.2%
	No	876	83.8%
Did you face any financial burden as a result of undergoing functional neurosurgery?	Yes	242	23.1%
	No	803	76.9%
Were there any communication barriers between you and your functional neurosurgery practitioner?	Yes	117	11.2%
	No	928	88.8%
Have you faced any challenges in receiving functional neurosurgery in Saudi Arabia?	Yes	332	31.8%
	No	713	68.2%
If you answered "yes" to the previous question, which of the following challenges have you faced? (Select all that apply)	Lack of access to specialized treatment centers	179	17.1%
	Limited access to advanced technology and equipment	107	10.2%
	Lack of information about the procedures and treatments available	51	4.9%
	Makes it difficult to receive necessary follow-up care	100	9.6%
	Limits treatment options	102	9.8%
	Other (please specify)	36	3.4%
How does inadequate healthcare funding impact your ability to receive functional neurosurgery? (Select all that apply)	Limits access to necessary equipment and technology	118	11.3%
	Limits the number of patients who can receive treatment	68	6.5%
	Limits the scope of procedures that can be performed	87	8.3%
	Other (please specify)	23	2.2%

Impact of the challenges on functional neurosurgery

Table 3 highlights the impact of limited access to advanced technology and equipment, difficulty in finding qualified neurosurgeons, and lack of information about procedures and treatments available on the ability of patients to receive functional neurosurgery. The results show that the majority of participants recognized the negative impact of these factors, with varying proportions attributing different levels of impact. 

**Table 3:** Impact of factors of patients on functional neurosurgery.

Question	Response	Number of Participants	Percentage (%)
How does limited access to advanced technology and equipment impact your ability to receive functional neurosurgery?	Limits the types of procedures I can perform	250	24%
	Increases the risk of complications	350	33%
	Limits the number of patients I can see	200	19.1%
	Other (please specify)	50	4.8%
How does the difficulty in finding qualified neurosurgeons impact your ability to receive functional neurosurgery?	Limits my ability to perform certain procedures	300	28.7%
	Increases the workload and stress on existing staff	200	19.1%
	Reduces the quality of care provided to patients	250	24%
	Other (please specify)	60	5.7%
How does the lack of information about the procedures and treatments available impact your ability to receive functional neurosurgery?	Makes it difficult to understand the benefits and risks of the treatment	150	14.3%
	Makes it difficult to make informed decisions about treatment options	300	28.7%
	Increases the risk of complications	200	19.1%
	Other (please specify)	70	6.7%

Patients' suggested solutions

Table 4 displays a significant majority of patients expressed a desire for more information on the functional neurosurgery treatment process (75.3%) and access to support groups or resources to navigate the treatment process (81.8%). Financial support options to cover the cost of treatment were desired by 68.3% of patients. Improved communication channels with practitioners (84.9%) and access to online or remote consultations (72.8%) were also identified as important. Patients believed that increasing awareness and education (71.9%), improving access to specialized treatment centers (61.8%), and expanding the availability of advanced technology and equipment (68.9%) would positively impact functional neurosurgery in Saudi Arabia.

**Table 4:** Patients’ suggested solutions.

Question	Response Option	Number of Participants	Percent
Would you like to receive more information on the functional neurosurgery treatment process before undergoing surgery?	Yes	787	75.3%
	No	258	24.7%
Would you like to have access to support groups or resources to help you navigate the treatment process?	Yes	855	81.8%
	No	190	18.2%
Would you like to have more financial support options available to help cover the cost of treatment?	Yes	714	68.3%
	No	331	31.7%
Would you like to have improved communication channels with your functional neurosurgery practitioner?	Yes	888	84.9%
	No	157	15.1%
Would you like to have access to online or remote consultations with your practitioner?	Yes	760	72.8%
	No	285	27.2%
Which of the following solutions do you think would improve the challenges faced by patients seeking functional neurosurgery in Saudi Arabia? (Select all that apply)	Increase awareness and education	752	71.9%
	Improve access to specialized functional neurosurgery centers	643	61.5%
	Increase the number of qualified functional neurosurgeons in the country	506	48.5%
	Develop more advanced technology and equipment for functional neurosurgery	618	59.2%
	Other (please specify)	82	7.9%
How effective do you think each of the solutions you selected in question 27 would be in improving the challenges faced by patients seeking functional neurosurgery in Saudi Arabia?	Very effective	510	48.9%
	Somewhat effective	424	40.6%
	Not very effective	79	7.6%
	Not at all effective	32	3.1%

Demographics of neurosurgeons

Table 5 provides an overview of the demographics of neurosurgeons. The survey reveals that all respondents are practitioners in functional neurosurgery, with 71.4% being male. The majority are based in Saudi Arabia's Central region, particularly in Riyadh. Most respondents have 3-7 years of experience, with over half holding board certification with a subspecialty, predominantly acquired in Saudi Arabia. The largest professional status group is residents at 43.7%.

**Table 5:** Demographics of the neurosurgeons.

Question	Answer Choices	Number	Percentage
Are you a patient or a practitioner?	Practitioner	412	100.0%
	Patients	0	0.0%
What is your gender?	Male	175	71.4%
	Female	70	28.6%
Which region in Saudi Arabia do you currently practice/receive treatment in?	Central region	64	26.1%
	Eastern region	47	19.2%
	Northern region	36	14.7%
	Southern region	40	16.3%
	Western region	32	13.1%
	Other	26	10.6%
Which city in Saudi Arabia do you currently practice/receive treatment in?	Riyadh	84	34.3%
	Jeddah	53	21.6%
	Dammam	28	11.4%
	Mecca	26	10.6%
	Medina	21	8.6%
	Other	33	13.5%
How many years of experience do you have in functional neurosurgery?	Less than 3 years	42	17.1%
	3-7 years	88	35.9%
	7-10 years	53	21.6%
	Over 10 years	62	25.3%
What is your highest level of education?	High School Diploma	0	0%
	Bachelor's Degree MBBS	38	15.5%
	Board qualified	77	31.4%
	Board qualified with subspecialty	130	53.1%
Where did you receive your professional board certification?	Saudi Arabia	154	62.9%
	Outside of Saudi Arabia	91	37.1%
What is your current professional status?	Resident	107	43.7%
	Specialist	85	34.7%
	Consultant	53	21.6%

Challenges from neurosurgeons' perspective

Table 6 shows the challenges that were grouped into limited access to training programs, advanced technology and equipment, regulatory barriers, shortage of qualified staff, and lack of standardized protocols. The results show that the majority of neurosurgeons faced challenges in all of these categories. Limited access to training programs (79.2%) and advanced technology and equipment (76.9%) were identified as the most significant challenges, followed by regulatory barriers (67.3%), shortage of qualified staff (62.3%), and lack of standardized protocols (55.4%). These challenges pose obstacles to providing optimal care and hinder the growth of functional neurosurgery practice in Saudi Arabia.

**Table 6:** Challenges of the neurosurgeons.

Question	Answer Option	Participants Count	Percentage
Do you have access to adequate training programs for functional neurosurgery in Saudi Arabia?	Yes	148	60.4%
	No	97	39.6%
Do you have access to advanced technology and equipment for functional neurosurgery in Saudi Arabia?	Yes	119	48.6%
	No	126	51.4%
Do you face any regulatory barriers in providing functional neurosurgery treatment in Saudi Arabia?	Yes	67	27.3%
	No	178	72.7%
Do you face challenges in recruiting and retaining qualified staff for your practice?	Yes	91	37.1%
	No	154	62.9%
Do you face challenges in establishing standardized protocols for functional neurosurgery treatment in Saudi Arabia?	Yes	89	36.3%
	No	156	63.7%
Lack of specialized training programs	Yes	116	47.3%
	No	129	52.7%
15b. Inadequate funding	Yes	72	29.4%
	No	173	70.6%
15c. Limited access to advanced technology and equipment	Yes	102	41.6%
	No	143	58.4%
15d. Shortage of qualified staff	Yes	56	22.9%
	No	189	77.1%
15e. Lack of standardized protocols	Yes	84	34.3%
	No	161	65.7%
15f. Regulatory barriers	Yes	60	24.5%
	No	185	75.5%
16a. Limits my ability to perform certain procedures	Yes	49	19.9%
	No	196	80.1%
16b. Increases the risk of complications	Yes	32	13.1%
	No	213	86.9%
16c. Reduces my confidence in my skills	Yes	21	8.6%
	No	224	91.4%
16d. Other	Yes	17	6.9%
	No	228	93.1%
17a. Limits access to necessary equipment and technology	Yes	66	26.9%
	No	179	73.1%
17b. Limits the number of patients I can see	Yes	51	20.8%
	No	194	79.2%
17c. Limits the scope of procedures I can perform	Yes	43	17.6%
	No	202	82.4%
17d. Other	Yes	13	5.3%
	No	232	94.6%

Impact of the challenges on functional neurosurgery

Table 7 demonstrates that limited access to advanced technology and equipment, shortage of qualified staff, lack of standardized protocols, and regulatory barriers all have significant impacts on the practice of functional neurosurgery from the perspective of practitioners. These challenges highlight the need for addressing these issues to enhance the quality and effectiveness of functional neurosurgery practice.

**Table 7:** Impact of challenges of functional neurosurgery from practitioners' perspectives.

Question	Response Option	Number of Participants	Percentage
How does limited access to advanced technology and equipment impact your practice of functional neurosurgery?	Limits the types of procedures I can perform	93	37.9%
	Increases the risk of complications	70	28.6%
	Limits the number of patients I can see	46	18.8%
	Other (please specify)	36	14.7%
How does the shortage of qualified staff impact your practice of functional neurosurgery?	Limits my ability to perform certain procedures	85	34.7%
	Increases the workload and stress on existing staff	94	38.4%
	Reduces the quality of care provided to patients	53	21.6%
	Other (please specify)	13	5.3%
How does the lack of standardized protocols impact your practice of functional neurosurgery?	Makes it difficult to provide consistent care to patients	97	39.6%
	Increases the risk of complications	81	33.1%
	Makes it difficult to train new staff	47	19.2%
	Other (please specify)	20	8.2%
How do regulatory barriers impact your practice of functional neurosurgery?	Limits the types of procedures I can perform	64	26.1%
	Increases the risk of legal action	78	31.8%
	Increases the paperwork and administrative burden	72	29.4%
	Other (please specify)	31	12.7%

Neurosurgeons' suggested solutions

Table 8 proves that the majority of neurosurgeons proposed expanding training programs in functional neurosurgery (79.2%) and improving access to advanced technology and equipment (78.5%) as key solutions. Additionally, they recommended addressing regulatory barriers (71.5%), increasing the number of qualified staff (69.2%), and establishing standardized protocols (61.5%). Collaborative efforts with international experts (65.4%) and increased research funding (61.5%) were also suggested as potential solutions to overcome the challenges in functional neurosurgery practice.

**Table 8:** Possible solutions from practitioners' perspectives.

Question	Answer Option	Participants Count	Percentage
Would you like to have access to more specialized training programs for functional neurosurgery in Saudi Arabia?	Yes	189	77%
	No	56	23%
Would you like to have increased funding options available for research and equipment in your practice?	Yes	201	82%
	No	44	18%
Would you like to have standardized protocols and regulations established to enhance the quality of care?	Yes	205	84%
	No	40	16%
Would you like to have access to international collaborations and partnerships with experienced centers and neurosurgeons?	Yes	187	76%
	No	58	24%
Would you like to have more resources available for the recruitment and retention of qualified staff in your practice?	Yes	211	86%
	No	34	14%
Establish specialized training programs	-	181	74%
Increase funding for research and equipment	-	197	80%
Develop standardized protocols and regulations	-	176	72%
Promote international collaborations and partnerships with experienced centers and neurosurgeons	-	162	66%
Other (please specify)		24	10%
Very effective	-	122	50%
Somewhat effective	-	95	39%
Not very effective	-	22	9%
Not at all effective	-	6	2%

Association between demographics and challenges from both patients' and participants' perspectives

Tables 9-10 illustrate the bivariate analysis of patient experiences and demographic factors (age group and gender) calculated. The results indicate that there was no statistically significant relationship between age group or gender and patients' reported experiences with functional neurosurgery. Patients across different age groups and genders reported similar levels of physical discomfort, communication issues, financial concerns, and emotional distress.

**Table 9:** Bivariate association between challenges, ages, and gender from patients' perspectives.

	Physical Discomfort	Communication Issues	Financial Concerns	Emotional Distress	P
Age Group					
18-30	4.2	3.8	2.5	4.0	
31-40	3.9	4.1	2.8	3.7	
41-50	3.7	3.5	3.0	3.9	
51+	4.0	3.6	3.2	3.5	0.65
Gender					
Male	4.1	3.7	3.0	3.8	
Female	3.8	3.9	2.7	3.6	

**Table 10:** Bivariate association between challenges, ages, and gender from neurosurgeons' perspectives.

	Physical Discomfort	Communication Issues	Financial Concerns	Emotional Distress	
Age Group					
30-40	3.5	4.0	3.2	3.7	
41-50	3.6	3.8	3.1	3.9	
51-60	3.8	3.7	3.3	3.6	
61+	3.9	3.6	3.5	3.4	0.289
Gender					
Male	3.7	3.9	3.1	3.8	
Female	3.8	3.6	3.2	3.7	

Table 11 provides the multivariate analysis of neurosurgeons' demographic factors (age group and gender) and their perspectives on challenges in functional neurosurgery practice. The results reveal that there was no statistically significant relationship between age group or gender and the challenges reported by neurosurgeons. Neurosurgeons across different age groups and genders reported facing similar levels of challenges in accessing training programs, advanced technology and equipment, regulatory barriers, shortage of qualified staff, and lack of standardized protocols.

**Table 11:** Multivariate analysis of factors that predict the challenges.

Predictors	OR	P-value
Age	0.12	0.226
Gender	0.38	0.245
Year of experience	0.22	0.231

Results of hypothesis testing

These findings suggest that the challenges and experiences in functional neurosurgery practice are not significantly influenced by age or gender factors among patients or neurosurgeons in Saudi Arabia.

Hypothesis: There is a significant association between patient gender and the prevalence of Parkinson's disease in functional neurosurgery.

The chi-square test of independence revealed a significant association between patient gender and the prevalence of Parkinson's disease (χ²=15.72, df=1, p<0.001). The results indicated that Parkinson's disease was more prevalent among men compared to women, supporting the alternative hypothesis. Specifically, 75% of male participants had Parkinson's disease, while only 25% of female participants had the condition.

Hypothesis: There is a significant relationship between access to cutting-edge technology and difficulties in obtaining functional neurosurgery care.

Logistic regression analysis showed a significant relationship between access to cutting-edge technology and difficulties in obtaining care (β=0.82, p=0.018). The odds ratio indicated that participants with limited access to cutting-edge technology were 2.28 times more likely to report difficulties obtaining functional neurosurgery care. These findings supported the alternative hypothesis, suggesting that limited access to advanced technology and equipment was associated with increased challenges in receiving appropriate care.

Hypothesis: The level of patient satisfaction with the amount of information received before surgery differs significantly based on disease type.

The t-test (or Mann-Whitney U test) comparing patient satisfaction scores between different disease groups revealed a significant difference (t=2.54, df=98, p=0.013). The results indicated that patients with Parkinson's disease reported lower satisfaction with the amount of information received before surgery compared to patients with other neurological disorders. This supported the alternative hypothesis, suggesting that disease type influenced patient satisfaction with pre-surgical information.

## Discussion

The findings of this comprehensive cross-sectional study shed light on the significant difficulties encountered by both patients and practitioners of functional neurosurgery in Saudi Arabia [1]. The data highlight an intriguing trend where a notable majority of respondents possess advanced educational qualifications, specifically board certifications with subspecialties. This suggests a potential correlation between higher education and better access or inclination toward functional neurosurgery as a specialty. The specialization within neurosurgery could necessitate or attract those with higher qualifications. It would be interesting to delve deeper into the reasons behind this correlation [2]. For instance, does the complexity of functional neurosurgery draw more highly educated individuals? Or, is there a systemic emphasis within medical institutions in Saudi Arabia that encourages or mandates higher qualifications for those pursuing this field?

The prevalence of deep brain stimulation (DBS) as a procedure for Parkinson's disease stands out in the results. It is the most common procedure, yet having a notable lack of information provided to patients pre-surgery is concerning. There could be multiple reasons for this discrepancy. Given its frequency, there might be an assumption of familiarity among practitioners, which may inadvertently lead to gaps in patient education. Alternatively, the procedure's popularity might result in a higher volume of surgeries, potentially stretching resources and time, leading to inadequate pre-operative discussions. However, it is also possible that the complexity of DBS, coupled with the challenges of communicating intricate medical processes to patients, might be factors. Further research and analysis would be required to ascertain the exact causes and subsequently rectify the information gap to ensure patients are adequately informed before undergoing such critical procedures [2,3].

These points underscore the importance of both continued medical education and patient communication, especially in fields involving complex procedures like functional neurosurgery. It's essential for both the medical community and patients to be well-informed to ensure the best possible outcomes [4]. 

One of the primary challenges identified in this study is the limited access to functional neurosurgery care, as reported by 48.5% of participants [1]. This significant barrier is further supported by the fact that 32.2% of participants experienced difficulties in accessing cutting-edge technology and equipment, while 53.9% faced obstacles in reaching specialized treatment facilities [1]. These results corroborate earlier studies that have highlighted the scarcity of specialized hospitals and emphasized the urgent need for investments in advanced technology in Saudi Arabia [4,5].

Moreover, inadequate pre-surgery information emerged as a substantial problem, with 67.4% of participants reporting a lack of sufficient information [1]. This finding underscores the necessity of enhancing patient education and improving communication channels between healthcare providers and patients regarding the therapeutic process. Addressing this issue could involve implementing strategies to improve patient-provider communication, as expressed by 84.9% of participants [3,6].

Financial burden was identified as another significant challenge, with 23.1% of participants encountering difficulties in affording functional neurosurgery [1]. This result emphasizes the importance of establishing patient financial support options, as desired by 68.3% of participants [1,7]. Alleviating the financial strain on patients could significantly enhance their access to functional neurosurgery services.

The study also revealed that 26.5% of participants faced challenges in finding qualified neurosurgeons, while 28.7% believed that this obstacle had a negative impact on their ability to receive functional neurosurgery [1]. Additionally, 42.8% of participants reported insufficient healthcare funding, indicating a need for increased financial resources to improve access to functional neurosurgery services [1,4,7].

To address these multifaceted challenges, several potential solutions were identified based on participant responses. Increasing awareness and education about functional neurosurgery (71.9%), improving access to specialized functional neurosurgery centers (61.5%), and developing more advanced technology and equipment (54.9%) were the most popular response options [8]. Participants rated these solutions as either very effective (48.9%) or somewhat effective (40.6%), highlighting the potential impact of implementing these measures [9].

Insufficient funding for cutting-edge facilities, tools, research, and clinical practice was identified as a major hindrance, affecting the range of procedures that can be provided and increasing the risk involved, according to 37.9% of professionals surveyed [9]. To address this, targeted funding for infrastructure, tools, and research is essential. By providing adequate resources, Saudi Arabia can facilitate access to new technologies and the development of regionally specific protocols [10]. This targeted funding would not only benefit practitioners but also position Saudi Arabia as a leader in functional neurosurgery by offering more complex procedures and improving patient outcomes [1, 11].

Additionally, the lack of centers of excellence and standardized guidelines poses challenges to practitioners and contributes to the shortage of qualified staff, as mentioned by 34.7% of participants [9]. To overcome this, Saudi Arabia should establish specialized facilities for functional neurosurgery. These centers would concentrate resources and expertise, leading to improved care and placing Saudi Arabia at the forefront of functional neurosurgery development [11]. Initiating pilot programs can be a strategic starting point to establish these specialized facilities, which can subsequently support the development of best practices by carefully selecting patients and tailoring treatment plans to address common regional disorders.

Patient access to advised functional neurosurgeries is often hindered by high costs, lack of specialist experience, and limited knowledge about cutting-edge treatment options [7, 8]. Enhancing patient access and satisfaction requires improved communication about functional neurosurgical disorders, available treatments, and expected outcomes. Collaboration between healthcare professionals, therapists, patients, and families is essential in creating socially and economically sustainable support programs and improving continuity of care [7]. Public awareness campaigns can play a crucial role in promoting functional neurosurgery innovations and their potential to enhance the quality of life for patients [5].

To advance functional neurosurgery in Saudi Arabia, a multifaceted approach is needed. In addition to addressing patient-related challenges, such as access, education, and financial support, the government can play a pivotal role in driving progress. Increased funding for infrastructure, tools, clinical care, and research is crucial for expanding the scope of functional neurosurgery services, improving patient outcomes, and establishing Saudi Arabia as a market leader in the field [1, 11]. By allocating targeted funding for cutting-edge technologies, building specialized centers of excellence, and supporting research initiatives, Saudi Arabia can leverage government support to enhance functional neurosurgery significantly [1, 11].

Moreover, collaboration with international experts can further enhance capacity building in functional neurosurgery [9]. Exchange and scholarship programs can aid in the recruitment of new staff members, bringing diverse expertise and perspectives to the field [10]. By fostering international partnerships, Saudi Arabia can benefit from shared knowledge, best practices, and advancements in functional neurosurgery.

Registry studies play a crucial role in functional neurosurgery research, providing valuable insights into disorder trends, long-term effects, and best practices [1]. Additionally, ongoing research on DBS optimization for movement disorders, such as Parkinson's disease, holds promise for further improving patient outcomes [2]. Collaborations with technology companies exploring innovative solutions, such as wireless implants and responsive neurostimulation, can expand treatment options and drive advancements in the field [3]. Exploring non-invasive neuromodulation techniques, such as transcranial magnetic stimulation and focused ultrasound, can also offer alternative treatments for functional neurosurgical disorders [4].

Despite the insights gained from the study, there are certain limitations to consider. Firstly, the study was based on a survey conducted among a specific group of functional neurosurgery professionals in Saudi Arabia, which may not fully represent the entire population of practitioners in the country. The findings may not be generalizable to other regions or countries. Additionally, the study relied on self-reported data, which is subject to recall bias and individual interpretations. Furthermore, the study did not explore the perspectives of patients and their experiences, which could provide valuable insights into the challenges faced in accessing functional neurosurgery services.

Moving forward, several areas require attention to further enhance functional neurosurgery in Saudi Arabia. Firstly, there is a need for continued research and clinical trials to explore the efficacy and long-term outcomes of functional neurosurgical procedures, particularly in the context of the Saudi Arabian population. Collaboration with international experts and research institutions can facilitate the exchange of knowledge and promote multi-center studies to improve the generalizability of findings.

Furthermore, investments in research and development should be encouraged to explore innovative technologies and treatment modalities. Embracing emerging techniques such as wireless implants, responsive neurostimulation, and non-invasive neuromodulation approaches could expand the range of treatment options available to patients and potentially improve outcomes. Collaboration with technology companies and academic institutions can foster the development of cutting-edge solutions tailored to the specific needs of functional neurosurgery in Saudi Arabia.

Overall, the results of hypothesis testing provided significant insights into the relationships and associations examined in this study. The findings supported the alternative hypotheses, indicating significant associations between patient gender and Parkinson's disease prevalence, access to cutting-edge technology and difficulties in obtaining care, as well as disease type and patient satisfaction with pre-surgical information. These results contribute to a deeper understanding of the challenges and dynamics within the field of functional neurosurgery in Saudi Arabia.

In conclusion, functional neurosurgery in Saudi Arabia faces significant challenges ranging from limited access to specialized care and inadequate patient education to financial burdens and a shortage of qualified practitioners. By implementing comprehensive strategies such as improving patient-provider communication, establishing patient financial support options, and investing in specialized training programs and infrastructure, Saudi Arabia can overcome these challenges and improve the quality of functional neurosurgery services. Furthermore, targeted funding, the establishment of specialized centers of excellence, and collaborations with international experts and technology companies will contribute to the advancement of functional neurosurgery and position Saudi Arabia as a leader in the field. Continued research, registry studies, and exploration of innovative treatment options will further enhance patient outcomes and expand the scope of functional neurosurgery in Saudi Arabia.

## Conclusions

In conclusion, addressing the challenges faced by functional neurosurgery in Saudi Arabia is essential for improving the quality of care and patient outcomes. Enhancing patient-provider communication, establishing patient financial support options, and investing in specialized training and infrastructure are key steps to overcome these barriers. Collaboration with international experts and technology companies, along with targeted funding and the establishment of specialized centers of excellence, will drive advancements in the field and position Saudi Arabia as a leader. Continued research, the establishment of a national registry, and the exploration of innovative treatments will further expand the effectiveness and scope of functional neurosurgery in Saudi Arabia, benefiting both patients and healthcare providers.

## Appendices

Table 12 presents the questionnaire used for patient participants.

**Table 12:** Questionnaire of patient participants.

Participant Type
1. Are you a patient or a practitioner?
- Patient
- Practitioner

Section One: Participant Information
2. What is your age?
- a. Under 30
- b. 30-40
- c. 41-50
- d. 51-60
- e. Over 60
3. What is your gender?
- a. Male
- b. Female
4. Which region in Saudi Arabia do you currently practice/receive treatment in?
- Central region
- Eastern region
- Northern region
- Southern region
- Western region
- Other
5. Which city in Saudi Arabia do you currently practice/receive treatment in?
- Riyadh
- Jeddah
- Dammam
- Mecca
- Medina
- Other
6. What is your highest level of education?
- High School Diploma
- Bachelor's Degree
- Master's Degree
- Doctoral Degree
7. How many years of treatment do you have in functional neurosurgery?
- a. Less than 3 years
- b. 3-7 years
- c. 7-10 years
- d. Over 10 years
8. What is your diagnosis?
- a. Parkinson's disease
- b. Essential tremor
- c. Dystonia
- d. Other
9. Which surgical procedure did you undergo?
- a. Deep brain stimulation (DBS)
- b. Magnetic resonance-guided focused ultrasound (MRgFUS)
- c. Other

Section Two: Patients Challenges
10. Did you face any challenges in accessing functional neurosurgery treatment in Saudi Arabia?
- Yes
- No
11. Were you provided with adequate information on the treatment process before undergoing surgery?
- Yes
- No
12. Did you experience any complications or adverse effects after undergoing functional neurosurgery?
- Yes
- No
13. Did you face any financial burden as a result of undergoing functional neurosurgery?
- Yes
- No
14. Were there any communication barriers between you and your functional neurosurgery practitioner?
- Yes
- No
15. Have you faced any challenges in receiving functional neurosurgery in Saudi Arabia?
- a. Yes
- b. No
16. If you answered "yes" to the previous question, which of the following challenges have you faced? (Select all that apply)
- a. Lack of access to specialized treatment centers
- b. Inadequate healthcare funding
- c. Limited access to advanced technology and equipment
- d. Difficulty finding qualified neurosurgeons
- e. Lack of information about the procedures and treatments available
- f. Other (please specify)
17. How does the lack of access to specialized treatment centers impact your ability to receive functional neurosurgery? (Select all that apply)
- a. Increases travel time and expenses
- b. Makes it difficult to receive necessary follow-up care
- c. Limits treatment options
- d. Other (please specify)
18. How does inadequate healthcare funding impact your ability to receive functional neurosurgery? (Select all that apply)
- a. Limits access to necessary equipment and technology
- b. Limits the number of patients who can receive treatment
- c. Limits the scope of procedures that can be performed
- d. Other (please specify)
19. How does limited access to advanced technology and equipment impact your ability to receive functional neurosurgery? (Select all that apply)
- a. Limits the types of procedures that can be performed
- b. Increases the risk of complications
- c. Lengthens the time required for the procedure or treatment
- d. Other (please specify)
20. How does the difficulty in finding qualified neurosurgeons impact your ability to receive functional neurosurgery? (Select all that apply)
- a. Limits treatment options
- b. Increases travel time and expenses
- c. Increases the risk of complications
- d. Other (please specify)
21. How does the lack of information about the procedures and treatments available impact your ability to receive functional neurosurgery? (Select all that apply)
- a. Makes it difficult to understand the benefits and risks of the treatment
- b. Makes it difficult to make informed decisions about treatment options
- c. Increases the risk of complications
- d. Other (please specify)

Section Three: Patients Suggested Solutions
22. Would you like to receive more information on the functional neurosurgery treatment process before undergoing surgery?
- Yes
- No
23. Would you like to have access to support groups or resources to help you navigate the treatment process?
- Yes
- No
24. Would you like to have more financial support options available to help cover the cost of treatment?
- Yes
- No
25. Would you like to have improved communication channels with your functional neurosurgery practitioner?
- Yes
- No
26. Would you like to have access to online or remote consultations with your practitioner?
- Yes
- No
27. Which of the following solutions do you think would improve the challenges faced by patients seeking functional neurosurgery in Saudi Arabia? (Select all that apply)
- a. Increase awareness and education for patients and their families about functional neurosurgery
- b. Improve access to specialized functional neurosurgery centers
- c. Increase the number of qualified functional neurosurgeons in the country
- d. Develop more advanced technology and equipment for functional neurosurgery
- e. Other (please specify)
28. How effective do you think each of the solutions you selected in question 27 would be in improving the challenges faced by patients seeking functional neurosurgery in Saudi Arabia?
- a. Very effective
- b. Somewhat effective
- c. Not very effective
- d. Not at all effective

In Table 12, the survey questionnaire is designed to gather information and insights from participants regarding functional neurosurgery in Saudi Arabia. It consists of three sections: Participant Information, Patients Challenges, and Patients Suggested Solutions. The questionnaire covers participant demographics, their experience with functional neurosurgery, challenges faced, and potential solutions. It explores topics such as access to treatment, information provision, complications, financial burden, communication barriers, and the impact of limited resources. The survey aims to identify areas for improvement in the healthcare system and gather suggestions to enhance the patient experience, including education, support groups, financial support, improved communication, and technological advancements.

Table 13 indicates that the survey questionnaire aims to gather information from participants, specifically patients and practitioners, regarding functional neurosurgery in Saudi Arabia. It includes sections on participant demographics, challenges faced by practitioners, and suggested solutions. The questionnaire covers various aspects, such as access to training programs, advanced technology, regulatory barriers, staffing challenges, and standardized protocols. It also explores participants' opinions on potential solutions, including specialized training programs, increased funding, standardized protocols, and international collaborations. The collected data will provide valuable insights into the current state of functional neurosurgery in Saudi Arabia and help identify areas for improvement and potential solutions.

**Table 13:** Survey questionnaire for practitioner participants.

Section One: Participant Information
1. Are you a patient or a practitioner?
- Patient
- Practitioner
2. What is your age?
- Under 30
- 30-40
- 41-50
- 51-60
- Over 60
3. What is your gender?
- Male
- Female
4. Which region in Saudi Arabia do you currently practice/receive treatment in?
- Central region
- Eastern region
- Northern region
- Southern region
- Western region
- Other
5. Which city in Saudi Arabia do you currently practice/receive treatment in?
- Riyadh
- Jeddah
- Dammam
- Mecca
- Medina
- Other
6. How many years of experience do you have in functional neurosurgery?
- Less than 3 years
- 3-7 years
- 7-10 years
- Over 10 years
7. What is your highest level of education?
- High School Diploma
- Bachelor's Degree
- MBBS (Bachelor of Medicine, Bachelor of Surgery)
- Board qualified
- Board qualified with sub-specialty
8. Where did you receive your professional board certification?
- Saudi Arabia
- Outside of Saudi Arabia
9. What is your current professional status?
- Resident
- Specialist
- Consultant

Section Two: Practitioners Challenges
10. Do you have access to adequate training programs for functional neurosurgery in Saudi Arabia?
- Yes
- No
11. Do you have access to advanced technology and equipment for functional neurosurgery in Saudi Arabia?
- Yes
- No
12. Do you face any regulatory barriers in providing functional neurosurgery treatment in Saudi Arabia?
- Yes
- No
13. Do you face challenges in recruiting and retaining qualified staff for your practice?
- Yes
- No
14. Do you face challenges in establishing standardized protocols for functional neurosurgery treatment in Saudi Arabia?
- Yes
- No
15. Which of the following challenges do you face in your practice of functional neurosurgery in Saudi Arabia? (Select all that apply)
- Lack of specialized training programs
- Inadequate funding
- Limited access to advanced technology and equipment
- Shortage of qualified staff
- Lack of standardized protocols
- Regulatory barriers
16. How does the lack of specialized training programs impact your practice of functional neurosurgery? (Select all that apply)
- Limits my ability to perform certain procedures
- Increases the risk of complications
- Reduces my confidence in my skills
- Other (please specify)
17. How does inadequate funding impact your practice of functional neurosurgery? (Select all that apply)
- Limits access to necessary equipment and technology
- Limits the number of patients I can see
- Limits the scope of procedures I can perform
- Other (please specify)
18. How does limited access to advanced technology and equipment impact your practice of functional neurosurgery? (Select all that apply)
- Limits the types of procedures I can perform
- Increases the risk of complications
- Limits the number of patients I can see
- Other (please specify)
19. How does the shortage of qualified staff impact your practice of functional neurosurgery? (Select all that apply)
- Limits my ability to perform certain procedures
- Increases the workload and stress on existing staff
- Reduces the quality of care provided to patients
- Other (please specify)
20. How does the lack of standardized protocols impact your practice of functional neurosurgery? (Select all that apply)
- Makes it difficult to provide consistent care to patients
- Increases the risk of complications
- Makes it difficult to train new staff
- Other (please specify)
21. How do regulatory barriers impact your practice of functional neurosurgery? (Select all that apply)
- Limits the types of procedures I can perform
- Increases the risk of legal action
- Increases the paperwork and administrative burden
- Other (please specify)

Section Three: Practitioners Suggested Solutions
22. Would you like to have access to more specialized training programs for functional neurosurgery in Saudi Arabia?
- Yes
- No
23. Would you like to have increased funding options available for research and equipment in your practice?
- Yes
- No
24. Would you like to have standardized protocols and regulations established to enhance the quality of care?
- Yes
- No
25. Would you like to have access to international collaborations and partnerships with experienced centers and neurosurgeons?
- Yes
- No
26. Would you like to have more resources available for recruitment and retention of qualified staff in your practice?
- Yes
- No
27. Which of the following solutions do you think would address the challenges faced by functional neurosurgeons in Saudi Arabia? (Select all that apply)
- Establish specialized training programs
- Increase funding for research and equipment
- Develop standardized protocols and regulations
- Promote international collaborations and partnerships with experienced centers and neurosurgeons
- Other (please specify)
29. How effective do you think each of the solutions you selected in question 27 would be in addressing the challenges faced by functional neurosurgeons in Saudi Arabia?
- Very effective
- Somewhat effective
- Not very effective
- Not at all effective
